# Pathological, molecular, and serological study of small ruminant lentiviruses in Jordan

**DOI:** 10.14202/vetworld.2022.1423-1429

**Published:** 2022-06-09

**Authors:** Nabil Q. Hailat, Tameem B. Algharaibeh, Laith N. Al-Eitan

**Affiliations:** 1 Department of Veterinary Pathology and Public Health, Jordan University of Science and Technology, Irbid, Jordan; 2 Department of Biotechnology and Genetic Engineering, Jordan University of Science and Technology, Irbid, Jordan

**Keywords:** caprine arthritis encephalitis virus, enzyme-linked immunosorbent assay, histopathology, maedi-visna virus, polymerase chain reaction, small ruminant lentiviruses

## Abstract

**Background and Aim:**

Maedi-visna is a chronic viral disease of sheep with worldwide distribution causing substantial economic losses to the small ruminant industry. Pneumonia and mastitis are the main manifestations of the disease. This study aimed to investigate the occurrence of maedi-visna virus (MVV) in sheep using histopathology and nested polymerase chain reaction (PCR) techniques and also to estimate the seroprevalence of small ruminant lentiviruses (SRLVs) in sheep and goats using commercially available enzyme-linked immunosorbent assay (ELISA).

**Materials and Methods:**

Lung tissue samples from 380 sheep were collected and fixed in 10% formalin for histopathology and molecular diagnosis of MVV. Separately, 806 serum samples were randomly collected from 633 sheep and 173 goats to detect the seroprevalence of SRLVs using ELISA.

**Results:**

The results showed that 4.7% of lung samples (n=190) were positive by both histopathology and nested PCR, 5.8% (n = 380) were positive by histopathology only (have lymphoid follicular hyperplasia), and 7.4% (n = 190) were positive by nested PCR only. Statistical analysis revealed a moderate agreement between the two tests (Kappa=0.451, n = 190). Serology results revealed that sheep and/or goats herd prevalence was 59.8% (n = 87), while individual seroprevalence in sheep (40.1%, n = 633) was significantly higher than that in the other six countries and also significantly higher than that in goats (18.5%, n = 173) (at p < 0.05).

**Conclusion:**

The moderate statistical agreement between nested PCR and histopathological diagnosis of MVV in formalin-fixed paraffin-embedded sheep lung tissue samples (Kappa=0.451, n = 190) suggests combining both tests for more sensitive MVV detection in sheep lung samples. SRLVs seropositivity in sheep was significantly higher than in goats, thus, it is of high concern and urges the inquiry into the economic impact of the disease and the financial benefit of adopting eradication measures.

## Introduction

The viruses in the small ruminant lentiviruses (SRLVs) group consist of a narrow phylogenetic extension of viral isolates with a relatively low genetic homogeneity. Viruses in this group have a positive sense single-stranded ribonucleic acid (RNA) genome [1] that represents viral genetic variants that are the cause of maedi-visna virus (MVV) in sheep and caprine arthritis encephalomyelitis virus (CAEV) in goats. Both diseases are mainly characterized by four well-known, relatively chronic, and slowly progressive pathologic outcomes: pneumonia, arthritis, encephalomyelitis, and mastitis [2–4]. Because seroconversion of infected animals requires a relatively long time (several months) [5], some infected animals remain undiagnosed, causing the disease to spread readily [6]. To avoid false-negative results of serological tests (due to the delay of seroconversion), it is suggested to combine polymerase chain reaction (PCR) with serological tests, as PCR can detect the nucleic acid of proviral particles which are present long before seroconversion [7]. Proviral particles are detectable in many body samples of infected animals. The earliest cellular concentration of proviral particles (the most reliable as well) is mononuclear cells of the buffy coat from peripheral blood samples [8]. The main route of virus transmission is colostrum and milk ingestion of newborns from their infected dams. In addition, horizontal transmission through secretions of the upper respiratory tract is also an important means of infection. In contrast, semen and transplacental transmission routes are also possible but with unspecified significance [3, 9]. A record of multispecies viral molecular detection of some SRLVs isolates and sheep/goat cross-species transmission has been experimentally demonstrated [10].

Furthermore, the demonstration of goat MVV/CAEV natural coinfection has been considered evidence of the possibility of the *in vivo* recombination [9, 11–13]. Respiratory signs are the most evident, including dyspnea and tachypnea, as interstitial pneumonia develops, with heavy lymphocytic infiltrates. Grossly, the lungs appear enlarged and fail to collapse, with multiple grey foci. Palpation reveals many firm areas, and mediastinal lymph nodes are markedly enlarged [14, 15]. Microscopically, changes in the lung consist of lymphoid follicular hyperplasia (LFH), diffuse thickening of alveolar septa and smooth muscle hyperplasia [16, 17], ok physical examination of the udder reveals diffuse nonpainful firmness. Milk production is markedly decreased. Histologically, lesions in the mammary gland are characterized as nonsuppurative nodular interstitial mastitis [3, 15]. Nervous signs depend on the severity of the case, including incoordination, paresis, hindlimb weakness, and ataxia in moderate cases. In contrast, total paralysis in more severe cases develops due to extensive demyelination or severe meningoencephalitis, microgliosis, and astrogliosis [3, 15]. A less common outcome of MVV in sheep is arthritis, primarily in the carpal and tarsal joints, and causing lameness. Histologically, synovial membrane of the affected joint is infiltrated by mononuclear cells and is associated with both fibrosis and necrosis [2, 3, 15]. Other body organs may also display changes. In the liver of animals infected with MVV, there may be portal to portal bridging of lymphocytic aggregation with or without fibrosis [18]. Kidneys may be affected by membranoproliferative glomerulonephritis or interstitial nephritis [18]. Nodular or diffuse mononuclear infiltrates can be found in the heart [16].

A study conducted in Jordan [17] found that individual SRLVs seroprevalence values were 5.6% (n = 13) and 6.9% (n = 16) in sheep and 1% (n = 2) and 13.8% (n = 28) in goats depending on the targeted antibody, while herd SRLVs prevalence value was 78.6% (n = 22). A study conducted by Al-Qudah *et al*. [19] in 2006 found that individual CAEV seroprevalence was 8.9% (n = 98), while herd CAEV prevalence was 23.2% (n = 16). In Lebanon, a recent study showed that herd seroprevalence was 100% in Awassi sheep while individual prevalence was 71% [20].

This study compares histopathology and nested PCR diagnosis of MVV in abattoir harvested; formalin-fixed paraffin-embedded (FFPE) sheep lung tissue samples and investigates the seroprevalence of SRLVs in both sheep and goat species in the northern area of Jordan.

Veterinarians’ observation of sheep in durative mastitis in the field, the histopathology and molecular confirmation of some cases of at JUST Veterinary Health Centre, and the lack of recent studies about the seroprevalence in Jordan justify the conduct of this study that aims to investigate the occurrence of MVV in sheep using histopathology and PCR techniques and to update the seroprevalence of SRLVs in sheep and goats using commercially available enzyme-linked immunosorbent assay (ELISA).

## Materials and Methods

### Ethical approval

This study was approved by the Deanship of Research, Jordan University (approval number 127/2018). Collection of tissues was approved by slaughterhouse administration.

### Study period and location

The study was carried out from October 2018 to January 2020 in Northern part of Jordan. Lung tissue samples were collected from slaughterhouses in Mafraq, Irbid, and Ajloun governorates. Blood samples were collected during the same period from different villages in these governorates.

### Sample collection

Three hundred and eighty tissue samples of sheep lungs that were grossly suspected of MVV lesions were harvested by veterinary inspectors at three abattoirs in Irbid, Jerash, and Mafraq. Lung tissue samples were kept in 10% formalin for at least 24 h before embedding for histopathology and PCR procedures for MVV diagnosis. Separately, a total of 806 blood samples (633 sheep and 173 goats) representative of 87 local herds at 46 villages in the four northern governorates of Jordan (Ajloun, Irbid, Jerash, and Mafraq) were collected. Blood samples came from mixed hosts; sheep and goats, but usually, the population of goats was much smaller than that of sheep. The main breed of sheep was Awassi, and for goats was the local black. The four governorates are about 35 Km apart from each other. Blood samples (10 mL) were centrifuged at 1500× g to obtain sera, which were then kept frozen at −20°C until applying the ELISA procedure per plate.

### Histopathological examination

A routine histopathology technique was applied according to Suvarna *et al*. [21]. Briefly, this includes trimming lung tissue into cassettes, automated tissue processing, paraffin wax embedding, 5 μm thickness microtomy and slide mounting, oven then xylene deparaffinization, and H&E staining. Slides were examined by microscope to inspect the three main morphologic changes caused by MVV in the lung. Any remarkable lesions were also recorded using a spreadsheet.

### Nucleic acid preparation

Total RNA was isolated from 190 sheep lung FFPE tissue samples using RNeasy FFPE Kit (Qiagen, Cat no. 73504, Germany). Per isolation run, the eluted RNA concentrations and purities were measured and documented using a spectrophotometer (Jenway, Genova, Nano, UK) then reverse transcription (RT) step was performed according to PrimeScript™ RT Master Mix kit (TaKaRa Bio, Cat# RR036B, Japan). Complementary deoxyribonucleic acid was stored at −20°C until the time of PCR procedure.

### Nested PCR

Two consequent steps of PCR reactions were performed to detect the presence of MVV proviral particles by amplifying the consistent LTR region. Primers sets and thermocycler regimen were obtained as described by [22]. Products of the second PCR reactions were electrophoresed through a 1.5% TBE diluted agarose gel for 40 min at 110V. Gel documentation system (Enduro™ GDS, USA) relied on UV light illumination of the added 10 μL Ethidium Bromide (Sigma-Aldrich, USA) per 100 ml gel preparation.

### ELISA

The protocol of a commercial ELISA screening kit (ID Screen^®^ MVV/CAEV Indirect, product code: VISNAS-5P) was applied and validated according to the instructions of the manufacturer (IDVet, Innovative Diagnostic Montpellier, France).

### Statistical analysis

Kappa value was calculated using Statistical Package for the Social Sciences software version 16.0 (IBM Corp., NY, USA) and then indexed according to Thrusfield and Christley [23]. The statistical differences between seroprevalences were calculated using the Chi-square test (at p < 0.05). Fisher’s exact test was used when a proportion of < 5 was present in > 25% of the squares [24].

## Results

### Histopathological examination

**Figure-1:**
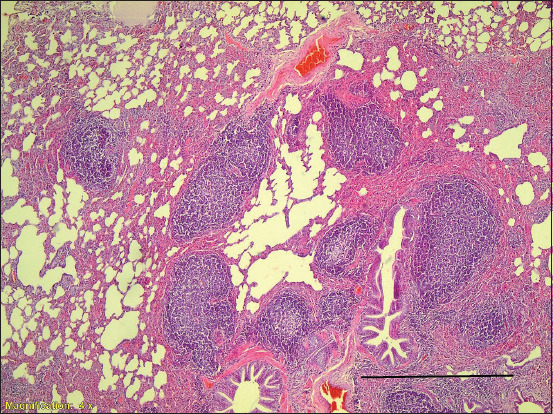
Lymphoid follicular hyperplasia with moderate to severe thickening of interalveolar septa. Sheep lung, Case M225, 40×, H and E stain. Scale Bar = 100um.

**Figure-2:**
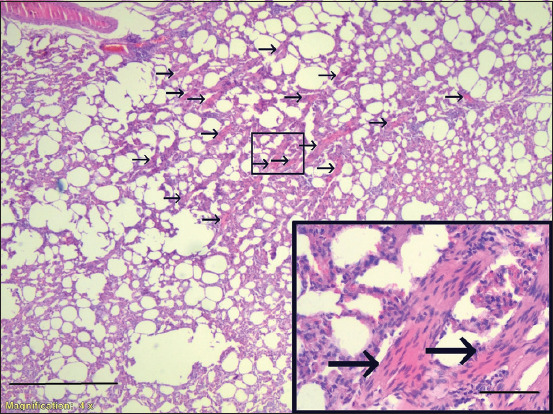
Smooth muscle hyperplasia, sheep lung, H&E stain, Case J57, 40× (Inserts are 400×). Scale Bar = 50 μm.

Histopathology revealed that 22 samples (5.8%) had LFH; the diagnostic lesion of MVV (Figure-1), 20 samples (5.3%) had smooth muscle hyperplasia (Figure-2), and 26 samples (6.8%) had alveolar septal thickening.

### Nested PCR

MVV targeted genome was detected in 14 (7.4%) samples (n = 190). Table-1 summarizes histopathological examination and nested PCR test results with fixation periods of each lung sample that had a positive result by at least one test. The results of histopathological and nested PCR diagnosis were tabulated, and Kappa value was calculated as 0.451 (Table-2).

**Table 1:** Summary of the results of the diagnostic techniques for the samples that had either MVV lung morphologic diagnosis or a positive nested PCR result with the fixation periods of each sample.

Sample number	Histopathology^1^			Nested PCR^2^	Fixation period (day)

	Lymphoid follicular hyperplasia (LFH)	Smooth muscle hyperplasia	Thickening of alveolar septa		
M35	−	−	−	+	2
M225^3^	+	−	+	+	3
M229	+	−	+	+	3
J57	+	+	+	+	3
M17	−	−	−	+	3
J235	+	+	+	+	4
J12	+	−	−	+	5
M42	−	−	−	+	5
M53	−	−	−	+	5
J199	+	−	−	−	8
M190	+	+	−	+	10
M240	+	−	−	+	10
M126	−	−	−	+	12
M205	+	−	−	+	13
M7	+	−	−	−	15
M11	+	−	+	−	15
M130	+	−	−	−	15
J169	+	−	−	−	15
M155	+	−	−	−	16
M230	+	−	−	−	16
I154	+	−	−	−	17
J59	+	−	−	+	17
J205	+	−	−	−	19
J209	+	−	−	−	19
J75	+	+	−	−	20
M56	+	+	−	−	21
J25	+	−	−	−	22

1. n = 380 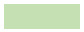 Only LFH positive

2. n = 190 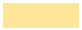 LFH & PCR positive

3. Detected by PCR and considered PCR positive control after confirmation by sequencing 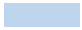 Only PCR positive

**Table 2:** Cross-tabulation of histopathology diagnosis (having lymphoid follicular hyperplasia) and nested PCR tests of Maedi-Visna Virus in sheep lung formalin-fixed paraffin-embedded tissue samples.

Histopathology	Nested PCR		Total

	Positive	Negative	
Positive	9	13	22
Negative	5	163	168
Total	14	176	190

PCR=Polymerase chain reaction

### ELISA

**Figure-3:**
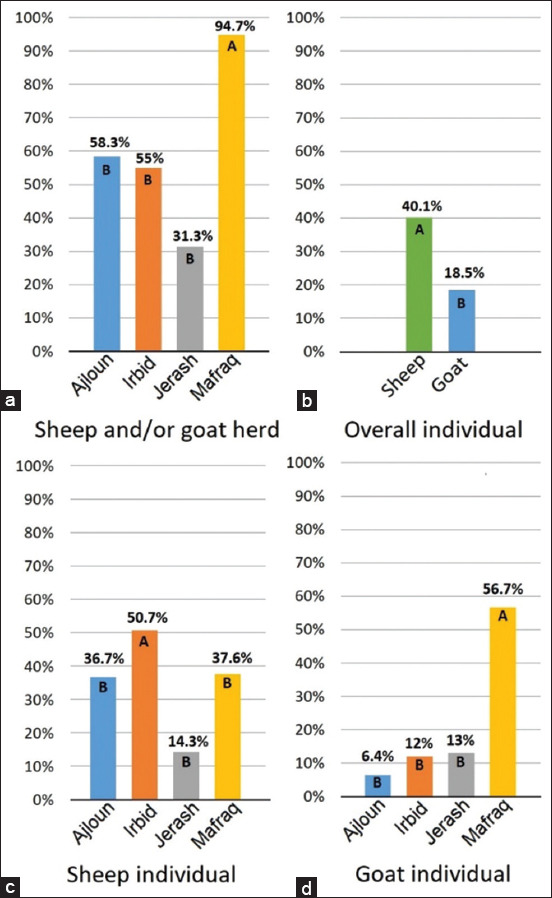
Bar diagrams of seroprevalences of small ruminant lentiviruses in northern governorates of Jordan. Letters indicate categorizations per statistically significant difference after applying Chi-square test for each match within each diagram (when the expected values of the cells were less than 5, Fisher’s exact test was used.) (a) Sheep and/or goat herd seroprevalence. (b) Governorates’ overall sheep and goat individual seroprevalence. (c) Sheep individual seroprevalence. (d) Goat individual seroprevalence.

The MVV/CAEV indirect ELISA screening test revealed a 59.8% (n = 87) sheep and/or goat herd seropositivity in northern governorates of Jordan (58.3% in Ajloun, n = 12; 55% in Irbid, n = 40; 31.3% in Jerash, n = 16; and 94.7% in Mafraq, n = 19). Mafraq herd seroprevalence was significantly higher than that of other governorates (Figure-3a). Individual seropositivity of sheep in the four governorates (40.1%, n = 633) was significantly higher than that of goat (18.5%, n = 173) (Figure-3b). Individual seropositivity of sheep in Irbid (50.7%, n = 136) was significantly higher than that in other governorates (36.7% [n = 30] in Ajloun, 14.3% [n = 7] in Jerash, and 37.6% [n = 460] in Mafraq) (Figure-3c). While individual seropositivity of goat in Mafraq (56.7%, n = 30) was significantly higher than that in other governorates (6.4% (n = 47) in Ajloun, 12% (n = 50) in Irbid, and 13% (n = 46) in Jerash) (Figure-3d).

## Discussion

**Figure-4:**
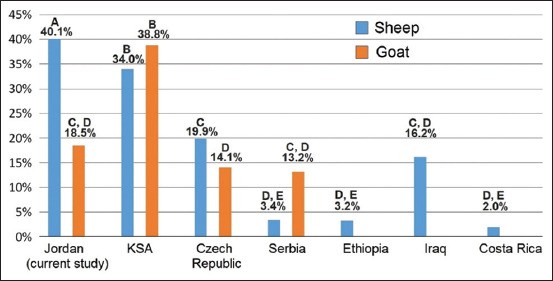
Bar diagram of individual seroprevalence of small ruminant lentiviruses for each of sheep and goats in the four northern governorates of Jordan (This study) and some studies in other countries using the same commercially available ID Screen Maedi-Visna Virus/caprine arthritis encephalomyelitis virus indirect enzyme-linked immunosorbent assay kit. (A, B, C, D, and E indicate categorization per statistically significant difference after applying Chi-square test at p < 0.05), A being the highest prevalence, followed by B, C, D, and E.

The histopathological result revealed that 22 (5.8%) FFPE lung tissue samples had LFH (the diagnostic lesion of MVV), only 9 of which had a positive PCR result. This disagreement between the results of those two tests may be attributed to false-negative results due to the decline in RNA quality caused by prolonged formalin fixation and/or tissue processing that affect RNA integrity and purity [24]. In our study, RNA purity and concentration were evaluated using spectrophotometry, but integrity has not been evaluated by any means. Table-1 shows that the 12 samples that had LFH with negative PCR result (92.3%, n = 13) were fixed with formalin for more than 14 days, and it is known that prolonged fixation in formalin can cause excessive breaks in nucleic acid, creating segments that are too short for the amplification procedure. While the eight samples that had LFH with positive PCR results (88.8%, n = 9) were fixed with formalin for <14 days. This finding is compatible with a previous research conducted by Benavides *et al*. [25] that studied the effect of fixation period on the amplification of MVV genome, which concluded that MVV nucleic acids are not detectable in lung tissue samples that were fixed for more than 14 days in formalin [25]. The statistical analysis of histopathological and nested PCR results found a Kappa value of 0.451, which indicates a moderate level of agreement between the two tests (n = 190) [23], and is consistent with a previous study suggesting that combining both of the tests is optimal for more sensitive MVV detection in sheep lung samples [26]. Although governorates’ overall individual seroprevalence in sheep (40.1%, n = 633) is significantly higher than that in goat (18.5%, n = 173) (Figure-3b), sheep individual seropositivity in Mafraq (37.6%, n = 460) falls under the lower significance category (Category B in Figure-3c), while that of goat (56.7%, n = 30) falls under the higher significance category (Category A in Figure-3d). Letters A and B indicate categorizations per statistically significant difference after applying Chi-square test for each match within each diagram in Figure-3 (when a proportion of < 5 was present in > 25% of the squares, Fisher’s exact test was used to calculate p-value rather Chi-square test, both tests at p < 0.05). These numbers and statistical significances may point to evidence that mixed sheep-goat herding practice is a crucial risk factor for harboring SRLVs. As sheep-goat contact in mixed-species herding is a risk factor of harboring SRLVs that was described by Lago *et al*. [27]. This risk factor was also demonstrated in a case–control study that found a relatively very high odds ratio of 26.9 of the presence of in-contact seropositive sheep to initiate a seroconversion in goat herds after three successive years of eradication [28]. Furthermore, this body of statistical evidence aligns with considerable clues debating that the phylogeny of the SRLVs isolates does not cluster them per host species [29]. Another important factor that might explain the significantly higher goat individual seropositivity than sheep in Mafraq governorate (Figure-3c and d) is the difference in herding practice between sheep and goats noted during sampling in that governorate. Sheep were herded to graze in open fields during daylight hours, while goats were kept inside narrow pens. This factor was demonstrated to be crucial for harboring the disease [30]. Furthermore, considering SRLVs as a viral genetic continuum that infects both sheep and goats remains a solid theoretical ground as a possible explanation to the statistical pattern of the occasional seropositivity that was described as “Tailing phenomenon” indicating the continuity of the seroprevalence line above zero on monitoring screening following up eradication programs [31]. Governorates’ overall individual seroprevalence in sheep (40.1%, n = 633) was significantly higher than that in goat (18.5%, n = 173). The results our study presented here reveal that SRLVs sheep individual seropositivity in northern governorates of Jordan (40.1%, n = 633) is significantly higher than that in other six countries where the same commercial indirect MVV/CAEV ELISA kit was used (3.4%, n = 2801 in Czech Republic [32], 34.0%, n = 147 in KSA [33], 19.9%, n = 3903 in Serbia [34], 3.2%, n = 494 in Ethiopia [35], 16.2%, n = 210 in Iraq [36], and 2%, n = 359 in Costa Rica [37]). SRLVs goat individual seropositivity (18.5%, n = 173) in northern governorates of Jordan was significantly lower than that in KSA (38.8%, n = 103) [33] and not significantly higher than that in Czech Republic (14.1%, n = 609) [32] and Serbia (13.2%, n = 1136) [34] (Figure-4). In Lebanon, the individual and herd seropositivity of Maedi in Awassi sheep were higher that the seropositivity in our study (71% and 100%, respectively) [20]. In Saskatchewan, Canada, the individual and herd seropositivity of maedi in sheep was much lower; 46% and 35%, respectively [38].

This commercial indirect ELISA screening test has never been used in Jordan before, but two previous studies screened sheep and goat sera for SRLVs seroconversion in Jordan [17, 19]. Those studies have used ELISA tests that detect sheep and goat antibodies against different MVV and CAEV proteins. Therefore, sensitivity and specificity variation between those different tests in the studies makes the comparison of the seropositivity of SRLVs in sheep and goat challenging to interpret. However, our finding that individual seropositivity is significantly higher in sheep than in goats agrees with the conclusion that most SRLVs in Jordan are MVV-like genotypes [17].

## Conclusion

The moderate statistical agreement between nested PCR and histopathological diagnosis of MVV in FFPE sheep lung tissue samples (Kappa = 0.451, n = 190) suggests combining both tests for more sensitive MVV detection in sheep lung samples. SRLVs seropositivity in sheep was significantly higher than in goats Our results raised high concern about the disease and urge the inquiry of the economic impact of the disease and the financial benefit of adopting eradication measures. Increased capacities and awareness of public and private veterinarians for proper recognition and diagnosis of MVV are needed to be listed in the disease priorities for control and prevention. In parallel, increased awareness of sheep and goat’s farmers of MVV are also needed and should be trained to recognize the disease based on clinical signs and finding when they slaughter animals on their own. This together can facilitate the adaptation of a control and prevention program by the Veterinary Services, at the Ministry of Agriculture, with emphasis on the control method appropriate for sheep farmers and the economy of the country. One of the limitations of such a control program is that it could not be obligatory because farmers can’t pay the cost of repeated testing and the adaptation of a new management plan to reduce seropositive animals and replace the flock from seronegative ewes offspring and culling of seropositive animals. The future plan is to propose a study using governmental sheep stations for the purpose of having maedi free flocks in Jordan. Furthermore, we plan to use immunohistochemistry on lung tissue samples to localize the distribution of the causative agent in the different components of the lung.

## Authors’ Contributions

NQH: Histopathological examination, results analysis, and writing proposal and manuscript. TBA: Sampling, histopathology procedure and examination, conducted ELISA and PCR, results analysis, visualization, and writing proposal and manuscript. LNA: Troubleshooting of PCR technique and helped in analysis of PCR results. All authors read and approved the final manuscript.
